# Using the Regression Slope of Training Loss to Optimize Chest X-ray Generation in Deep Convolutional Generative Adversarial Networks

**DOI:** 10.7759/cureus.77391

**Published:** 2025-01-13

**Authors:** Chih-Hsiung Chen, Kuang-Yu Hsieh, Kuo-En Huang, En-Tsung Cheng

**Affiliations:** 1 Department of Critical Care Medicine, Mennonite Christian Hospital, Hualien, TWN; 2 Department of Critical Care Medicine, Jen Ho Hospital, Show Chwan Health Care System, Changhua, TWN

**Keywords:** chest x-ray, deep convolutional generative adversarial networks, generative artificial intelligence, image generation, regression line, slope

## Abstract

Diffusion models, variational autoencoders, and generative adversarial networks (GANs) are three common types of generative artificial intelligence models for image generation. Among these, GANs are the most frequently used for medical image generation and are often employed for data augmentation in various studies. However, due to the adversarial nature of GANs, where the generator and discriminator compete against each other, the training process can sometimes end with the model unable to generate meaningful images or even producing noise. This phenomenon is rarely discussed in the literature, and no studies have proposed solutions to address this issue. Such outcomes can introduce significant bias when GANs are used for data augmentation in medical image training. Moreover, GANs often require substantial computational power and storage, adding to the challenges. In this study, we used deep convolutional GANs for chest X-ray generation, and three typical training outcomes were found. Two scenarios generated meaningful medical images and one failed to produce usable images. By analyzing the loss history during training, we observed that the regression line of the overall losses tends to diverge slowly. After excluding outlier losses, we found that the slope of the regression line within the stable loss segment indicates the optimal point to terminate training, ensuring the generation of meaningful medical images.

## Introduction

In recent years, artificial intelligence (AI) has been widely applied in medicine, spanning areas such as medical imaging diagnosis, disease prognosis prediction, and medical language modeling. Among the most promising fields are large language models (LLMs) and AI-assisted medical image analysis. LLMs, in particular, have gained widespread adoption with the introduction of publicly accessible models such as OpenAI's GPT [1], Google Gemini [2], Anthropic Claude [3], and Microsoft CoPilot [4], as well as open-source models like Meta's Llama [5] and Mistral AI, which offer fine-tuning tools for private datasets. These models are extensively used in healthcare for natural language processing and applications, even aiding triage in emergency departments [6]. Additionally, the new generation of LLMs has successfully passed not only general medicine exams [7] but also specialty board examinations for physicians [8].

In AI-assisted medical imaging analysis, the focus is primarily on diagnostics across modalities such as computed tomography (CT), magnetic resonance imaging (MRI), and, most commonly in critical care, chest X-rays (CXRs). Initial applications of CXRs were in detection tasks like tuberculosis screening [9], pneumonia diagnosis [10], and COVID-19 detection [11,12]. Since 2020, various applications in critical care medicine, such as cardiothoracic ratio measurement [13], endotracheal tube tip positioning [14], lung contusion detection [15], and acute respiratory distress syndrome identification [16], have achieved varying degrees of success.

Generative imaging represents one of the most promising branches of AI-assisted image processing. Leading generative models include OpenAI's DALL-E [17], MidJourney, Stability AI's Stable Diffusion [18], and Google DeepMind's Imagen [19], all of which have made significant breakthroughs in image generation. However, the application of generative AI to medical imaging has been relatively rare. The images produced by publicly accessible models are typically general illustrations, lacking the specificity needed for professional medical purposes. Moreover, these models do not currently offer fine-tuning capabilities. Due to concerns surrounding medical ethics, legalities, and liability, progress in professional medical image generation has been limited.

In response to the unique requirements of medical image generation, we developed a customized model to generate CXRs using a generative adversarial network (GAN) architecture. GAN-based generation demands significant computational power and storage, and determining the optimal number of training iterations remains challenging; even with extensive training, GANs sometimes fail to produce meaningful results or generate noisy images, which could hinder their application in medical imaging.

In our study, we found that monitoring training losses provided valuable insights. Specifically, by examining the slopes of the regression lines for the generator and discriminator losses, we identified an approach to determine an optimal stopping point for training. This approach, which has not been previously explored, can be utilized to optimize training termination by leveraging regression slopes. Integrating regression slope analysis into GAN training has the potential to significantly improve both efficiency and outcomes in medical imaging applications.

## Materials and methods

Dataset

In 2018, Daniel S. Kermany and his team from Guangzhou Women and Children’s Medical Center published a chest X-ray dataset intended for training AI in imaging applications. This dataset includes a total of 5,232 pediatric chest X-ray images, collected and annotated from 5,856 child patients. The ages of these patients range between one and five years. Among the images, 1,349 are labeled as normal, while the remaining 3,883 are labeled as pneumonic, consisting of 2,538 cases of bacterial pneumonia and 1,345 cases of viral pneumonia [20].

All images are classified into two categories: one for training/validation and the other for testing. In the TRAIN folder, there are 1,341 images labeled as normal and 3,875 labeled as pneumonia. The VAL set contains eight images labeled as normal and eight labeled as pneumonia. For testing purposes, a TEST subset from 624 patients was used, consisting of 234 images labeled as normal and 390 images labeled as pneumonia, of which 242 were bacterial and 148 were viral. In this study, we focused on generating normal CXR images. Therefore, only the 1,341 normal images from the TRAIN folder were selected.

Model architecture

Our primary architecture is inspired by Alec Radford, Luke Metz, and Soumith Chintala's deep convolutional generative adversarial networks (DCGANs) [21]. With some modifications, DCGAN introduced deeper layers that were organized into upsampling and downsampling blocks, along with a larger input image size capable of processing color information as a key feature.

Generator

The generator takes a latent vector as input and progressively upsamples it to generate a 256x256 grayscale image. It utilizes transposed convolution layers to increase the image resolution, with batch normalization and rectified linear unit (ReLU) activation to ensure stable training.

Initially, the latent vector is transformed into a tensor with a shape of 8x8x1024 and fed into the upsampling process after batch normalization and ReLU activation. Each upsampling block consists of a transposed convolution layer, a batch normalization layer, and ReLU activation applied to the block’s output. The process starts with the 8x8x1024 input, and the latent vector is upsampled through four blocks, producing feature maps with sizes 16x16x512, 32x32x256, 64x64x128, and 128x128x64. The final layer uses a transposed convolution to generate a 256x256x1 single-channel image, with a tanh activation function that constrains pixel values between -1 and 1. This generator is designed to create high-resolution images from random latent vectors.

Discriminator

The discriminator takes a 256x256 grayscale image as input and progressively downsamples it, eventually outputting a binary classification to indicate whether the input image is real or generated. The model employs convolutional layers for downsampling, paired with LeakyReLU activation and batch normalization for improved convergence stability.

The process begins with a 256x256x1 input image, which is first transformed into a tensor of size 128x128x32 through a convolution layer and LeakyReLU activation. The downsampling process then proceeds through four downsampling blocks, each consisting of a convolution layer, a batch normalization layer, and LeakyReLU activation. As the feature maps pass through the blocks, their sizes change from 128x128x32 to 64x64x64, 32x32x128, 16x16x256, and finally 8x8x512. The final feature map is flattened before being passed into a fully connected layer. The model uses a sigmoid activation function to output a binary classification, predicting whether the image is fake (denoted as 0) or real (denoted as 1). This discriminator is designed to effectively distinguish between real images and those generated by the generator (Table 1).

**Table 1:** The layers of generator and discriminator by TensorFlow.

Generator architecture				Discriminator architecture		
Layer (type)	Output shape	Param #		Layer (type)	Output shape	Param #
Dense	(None, 65536)	8454144		Conv2D	(None, 128, 128, 32)	800
BatchNormalization	(None, 65536)	262144		LeakyReLU	(None, 128, 128, 32)	0
ReLU	(None, 65536)	0		Conv2D	(None, 64, 64, 64)	51200
Reshape	(None, 8, 8, 1024)	0		BatchNormalization	(None, 64, 64, 64)	256
Conv2DTranspose	(None, 16, 16, 512)	13107200		LeakyReLU	(None, 64, 64, 64)	0
BatchNormalization	(None, 16, 16, 512)	2048		Conv2D	(None, 32, 32, 128)	204800
ReLU	(None, 16, 16, 512)	0		BatchNormalization	(None, 32, 32, 128)	512
Conv2DTranspose	(None, 32, 32, 256)	3276800		LeakyReLU	(None, 32, 32, 128)	0
BatchNormalization	(None, 32, 32, 256)	1024		Conv2D	(None, 16, 16, 256)	819200
ReLU	(None, 32, 32, 256)	0		BatchNormalization	(None, 16, 16, 256)	1024
Conv2DTranspose	(None, 64, 64, 128)	819200		LeakyReLU	(None, 16, 16, 256)	0
BatchNormalization	(None, 64, 64, 128)	512		Conv2D	(None, 8, 8, 512)	3276800
ReLU	(None, 64, 64, 128)	0		BatchNormalization	(None, 8, 8, 512)	2048
Conv2DTanspose	(None, 128, 128, 64)	204800		LeakyReLU	(None, 8, 8, 512)	0
BatchNormalization	(None, 128, 128, 64)	256		Conv2D	(None, 4, 4, 1)	12800
ReLU	(None, 128, 128, 64)	0		Flatten	(None, 16)	0
Conv2DTranspose	(None, 256, 256, 1)	1600		Activation	(None, 16)	0
Activation	(None, 256, 256, 1)	0		Total params: 4,369,440. Trainable params: 4,367,520. Non-trainable params: 1,920		
Total params: 26,129,728. Trainable params: 25,996,736. Non-trainable params: 132,992						

Training loss

The loss metric for the generator (g_loss) measures the binary cross-entropy distance between a tensor of ones (representing real labels) and the discriminator's output for fake images generated by the generator. When the g_loss is low, it indicates that the generator is producing images that appear more realistic to the discriminator. Conversely, if the g_loss is high, it suggests that the generator's images are easily recognized as fake by the discriminator.

The loss metric for the discriminator (d_loss) is the sum of the real loss and the fake loss. The real loss is the binary cross-entropy distance between the real labels (denoted as all ones in the tensor, slightly smoothed by a smooth factor to avoid overconfidence) and the discriminator’s output for real images. The fake loss is the binary cross-entropy distance between the fake labels (denoted as all zeros in the tensor) and the discriminator’s output for generated fake images.

Both the generator and discriminator are trained using their respective losses, g_loss and d_loss, which are adversarially optimized. The generator is trained to reduce its loss by producing increasingly realistic images that fool the discriminator into classifying them as real. The discriminator is trained to reduce its loss by improving its ability to distinguish between real and fake images.

Training and image generation

The code is based on TensorFlow 2.10 (Google, Mountain View, CA) running on a Windows platform with Python 3.10 (Python Software Foundation, Wilmington, Delaware). The training hardware is equipped with an NVIDIA RTX3060 6 GB graphics processing unit (GPU) and CUDA driver version 12.1 (NVIDIA, Santa Clara, CA). The training process was set for 500 epochs, with the following hyperparameters: latent dimension = 128, image height = 256, image width = 256, image channels = 1, batch size = 32, and kernel size = 5. The Adam optimizer was used with a learning rate of 0.00001 and beta_1 set to 0.5 for both the generator and discriminator. Keras’ built-in BinaryCrossentropy was employed as the loss function for both models.

Throughout the training process, generator and discriminator losses were recorded. We trained the DCGAN multiple times, summarizing the outcomes into three conditions. During training, we implemented a custom callback function that saved two sets of generator and discriminator weights as pre-trained models either at each epoch interval or at a specific epoch.

Using the trained generator, we produced CXR images. With the latent dimension set to 128 during training, we utilized the NumPy toolkit to randomly generate a matrix of 128 values as the input seed for the generator model. The generator then outputs a 256x256 grayscale image, which is saved and further processed with an image processing tool.

Regression of generator and discriminator losses

After training for 500 epochs, we used the numpy.polyfit function to fit a straight line to the generator and discriminator losses, obtaining the slope and intercept of the regression line. This regression trend analysis covers the losses from epoch 0 to epoch 500 to assess overall trends. Additionally, we perform a comparative analysis of specific intervals needed for the study, such as epochs 200 to 500 in result 1.

Slope of the regression line from the origin to a specific epoch

We examine the regression line's slope by setting the left endpoint at epoch 0 and varying the right endpoint to specific epochs 𝑛 (where 5≤𝑛<500), calculating the slope at each selected epoch 𝑛 and plotting an epoch-slope graph for further analysis. It is worth noting that if fewer than five data points are used for the regression, such as when the epoch count 𝑛 is below 5, the numpy.polyfit function may return a "poorly conditioned" error message. Therefore, for the overall epoch-slope graph, slope calculations begin from epoch 5.

For similar technical reasons, when focusing on specific intervals of loss trends, such as epochs 150 to 500 in result 2, the regression line is drawn starting at epoch 150, while the slope plot begins at epoch 155. In the case of result 3, the slope plot extends from epoch 155 to epoch 400. Potential trend inflection points in the loss plot were visually identified, approximately around epoch 150.

## Results

Image generation

**Figure 1:**
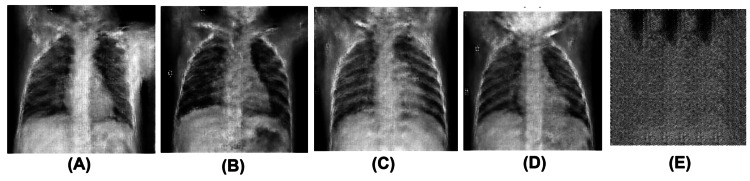
Fake chest X-ray images generated by the generator model. These images display structural features in real chest X-rays, such as the rib cage, symmetrical lung fields, diaphragm, left-sided heart, shoulders, arms, and neck. Image (A) shows a raised right arm and a lowered left arm, with a blurred and faint gastric bubble and trachea. Image (B) reveals a visible gastric bubble, a blurred tracheal shadow, and bilateral clavicles. Image (C) is notable for its remarkable heart size. Image (D) features a prominent blurred head shadow, resembling a pediatric chest X-ray (CXR). There are noticeable imperfections, such as slight blurring and artifacts throughout the images, which are common in generative adversarial network-generated outputs. Images (A) and (B) are CXR images generated using the generator model after 500 training epochs, while images (C) and (D) are images generated using the generator model after 200 training epochs. Image (E) produced no meaningful images after 500 epochs.

The generator model of the DCGAN uses an input noise vector of size 128. We generate a 128-dimensional noise vector using the Numpy toolkit to map random noise into realistic CXR images. The generated images are 256x256 pixels and grayscale. We used different random seeds to generate multiple CXRs, from which we selected four with distinct characteristics as the results, displayed in Figure 1.

These generated images resemble real CXRs and display certain characteristics typical of GAN-generated images, particularly regarding resolution and anatomical accuracy. While major anatomical structures such as the lung fields, diaphragm, rib cage, left-sided heart, neck, and arms are present, they lack fine details, clear anatomical outlines, and sharpness. Both lung fields exhibit a relatively symmetrical structure, but the edges of the ribs and the texture of the lung parenchyma appear blurred. Pixel-level inconsistencies or noise are evident, particularly in the upper areas near the shoulders and neck. Notable imperfections, including slight blurring and artifacts, are present throughout the images. This type of noise is commonly seen in synthetic images produced by GAN models or models trained on smaller datasets.

Generator and discriminator loss history

Result 1

The generator loss fluctuates significantly during the training of 500 epochs. The discriminator loss stays relatively low and stable throughout the course, which means the discriminator is doing well to distinguish real images from generated ones. The consistently low loss implies that the discriminator has become quite proficient at identifying fake images. The occasional spikes in the discriminator loss may coincide with moments when the generator produces images that are slightly better or worse, leading to brief confusion for the discriminator.

**Figure 2:**
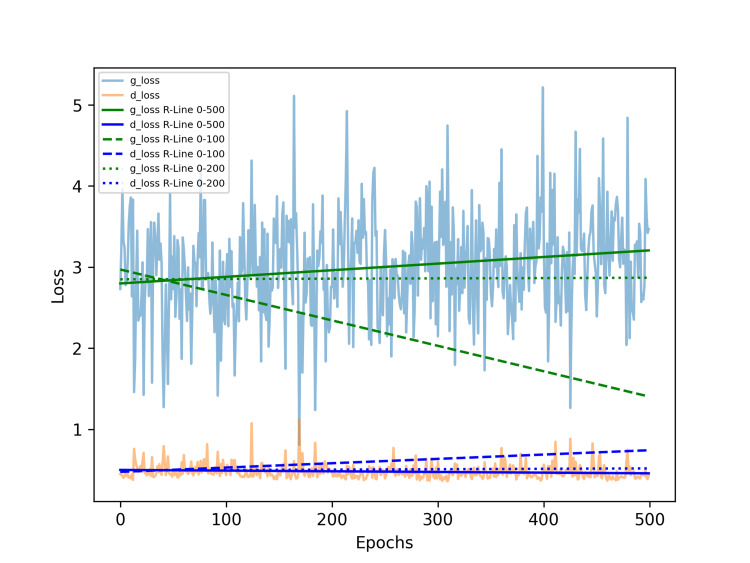
Loss curves and regression lines of the generator and discriminator during training. R-Line 0-500, R-Line 0-100, and R-Line 0-200 indicate regression lines based on losses over the entire training course of 500 epochs, the first 100 epochs, or the first 200 epochs. The green solid line represents the regression trend for the g_loss over 500 epochs, while the blue solid line represents the regression trend for the d_loss over the same period. The green dashed line represents the regression trend for g_loss during the first 100 epochs, and the blue dashed line represents the trend for d_loss over that same interval. Additionally, the green dotted line shows the regression trend for g_loss over the first 200 epochs, while the blue dotted line represents the corresponding trend for d_loss. The red dashed line indicates a gradual increase, whereas the orange dashed line shows a gradual decrease, suggesting an imbalance between the generator and discriminator during training. In contrast, the green dashed line reflects a gradual decrease, while the blue dashed line shows a gradual increase. Both the green and blue dotted lines remain nearly horizontal, suggesting that the optimal training point might occur around 200 epochs. Abbreviations: g_loss, generator loss; d_loss, discriminator loss; R-Line, regression line.

After 500 epochs, the descriptive statistical parameters for the generator model training loss were as follows: mean = 3.001, median = 3.020, standard deviation = 0.624, minimum = 0.806, and maximum = 5.214. For the discriminator model training loss, the parameters were: mean = 0.478, median = 0.457, standard deviation = 0.092, minimum = 0.356, and maximum = 1.127. We conducted a linear regression analysis on the generator and discriminator losses after training for 500 epochs, resulting in two linear equations: the generator loss is Y = 0.00082 X + 2.79763, and the discriminator loss is -0.00008 X + 0.49886, where Y represents the respective regression value of the loss, and X represents the epoch. These two lines were subsequently plotted on the loss history chart. We also conducted a linear regression analysis using the losses of the first 100 epochs of the generator and discriminator. The generator loss is Y = -0.00314 X + 2.96948, and the discriminator loss is Y = 0.00054 X + 0.47449. The equation of the first 200 epochs of the generator and discriminator is Y = 0.00004 X + 2.84875, and the discriminator loss is Y = 0.00005 X + 0.49271. Refer to Figure 2.

**Figure 3:**
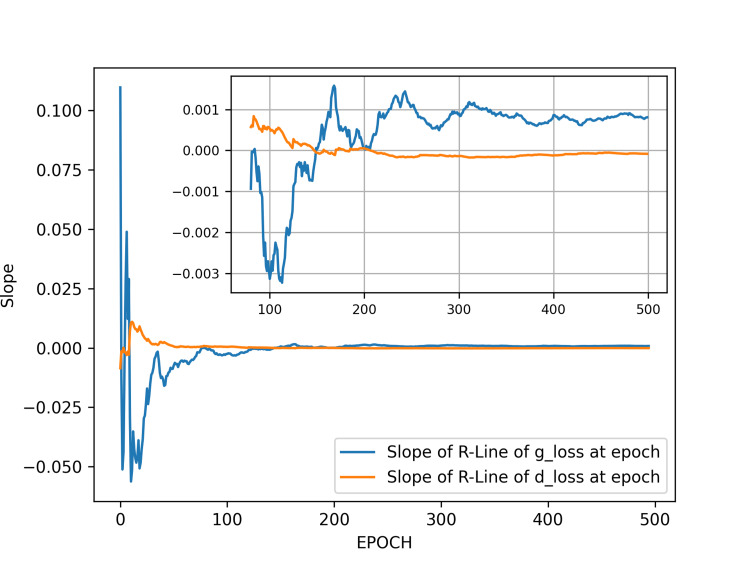
Slopes of the regression lines of generator and discriminator losses in result 1. The main plot shows the slopes over the epochs from five to 500. It is evident that the slopes of the regression lines fluctuate significantly at the beginning of training. However, as training progresses, the fluctuations gradually converge. The inset in the upper right corner highlights the range from epoch 80 to 500, where the slopes of the regression lines converge within a smaller range, indicating diminishing fluctuations. We selected generator models trained at epochs 200 and 500 for image generation comparison. Abbreviations: g_loss, generator loss; d_loss, discriminator loss; R-Line, regression line.

We next perform regression analysis on the g_loss and d_loss values from the range from the initial epoch to a specific epoch, plotting the two slope values of the two regression lines for the ranges of g_loss and d_loss. As seen, the training initially fluctuates significantly due to the adversarial nature of the GAN. However, as the number of training epochs increases, the slope of the overall regression curve begins to converge within a smaller range (Figure 3).

Result 2

During the initial training phase, the generator loss consistently increased until around 100-120 epochs, where it began to reverse and decrease. After 150 epochs, the generator loss approached a near-horizontal trend, fluctuating slightly within a narrow range for the remainder training course. In contrast, the discriminator loss remained relatively low and stable throughout the course following 150 epochs.

At the end of 500 epochs, the descriptive statistical parameters for the generator model's training loss were as follows: a mean of 4.063, median of 3.629, standard deviation of 1.223, minimum of 1.598, and maximum of 8.078. For the discriminator model's training loss, the parameters were: mean of 0.545, median of 0.548, standard deviation of 0.026, minimum of 0.501, and maximum of 0.807.

A linear regression analysis on the generator and discriminator losses after training for 500 epochs resulted in two linear equations: the generator loss is Y = -0.00391 X + 5.03948, and the discriminator loss is Y = 0.00004 X + 0.53467, where Y represents the respective regression value of the loss, and X represents the epoch. These two lines were subsequently plotted on the loss history chart. The equation of the first 100 epochs of the generator and discriminator is Y = 0.04636 X + 3.41604, and the discriminator loss is Y = -0.00079 X + 0.55893.

**Figure 4:**
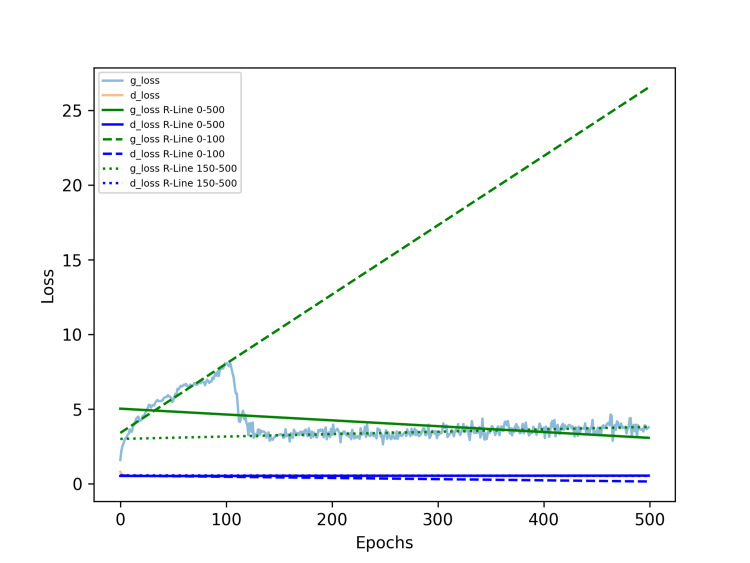
Loss curves and regression lines of the generator and discriminator in result 2. In the initial, the g_loss increases to the peak at 100-120 epochs and reverses to decrease. After the 150th epoch, the g_loss approached a near-horizontal trend and fluctuated within a narrow range for the rest course. The d_loss remained low and stable throughout the whole course. The green solid line represents the regression trend for the g_loss over 500 epochs, while the blue solid line represents the regression trend for the d_loss over the same period. The green dashed line represents the regression trend for g_loss during the first 100 epochs, and the blue dashed line represents the trend for d_loss over that same interval. The green dotted line shows the regression trend for g_loss over the first 200 epochs, while the blue dotted line represents the corresponding trend for d_loss during that period. The red dashed line indicates a gradual increase, whereas the orange dashed line shows a gradual decrease. In contrast, the green dashed line reflects a gradual decrease, while the blue dashed line shows a gradual increase, meaning progressive improvement. Both the green and blue dotted lines remain nearly horizontal, suggesting that the optimal training point might occur around 200 epochs. Abbreviations: g_loss, generator loss; d_loss, discriminator loss; R-Line, regression line.

However, due to the extreme values at the beginning, we believe that only the stabilized training values are meaningful for reference. We conducted a linear regression analysis using the generator and discriminator losses from 155 epochs onward until the end of training. The generator loss is Y = 0.00160 X + 3.01457, and the discriminator loss is Y = -0.00007 X + 0.57486. Refer to Figure 4.

**Figure 5:**
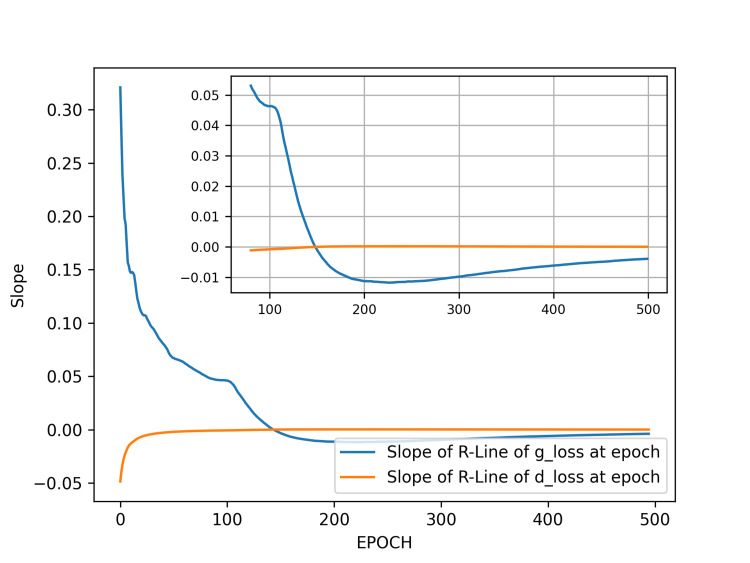
Slopes of the regression lines for generator and discriminator losses in result 2. The plot illustrates the slopes over epochs ranging from five to 500. The slope initially drops sharply from a positive value to a negative one, reaching its lowest point around epoch 150 before gradually approaching zero in the later training course. As seen in Figure 4, the g_loss becomes nearly horizontal after 150 epochs, fluctuating slightly within a narrow range for the remainder of the training. This trend arises mainly due to an initial upward trend in the losses between epochs 100 and 120, which, when included in the analysis, contrasts with the subsequent loss behavior. Abbreviations: g_loss, generator loss; d_loss, discriminator loss; R-Line, regression line.

We then performed a regression analysis on the g_loss and d_loss values from the initial epoch up to a specified epoch, plotting the slope values of the regression lines for both g_loss and d_loss ranges. Unlike the results in result 1, the training slope initially decreases steadily into negative values, reaching its lowest point between epochs 200-250, after which it gradually rises, nearing zero values. This trend, which does not align with the later d_loss values, occurs because the slope in Figure 5 is derived from cumulative regression starting from epoch 0, revealing a persistent upward trend in the first 100-120 epochs.

**Figure 6:**
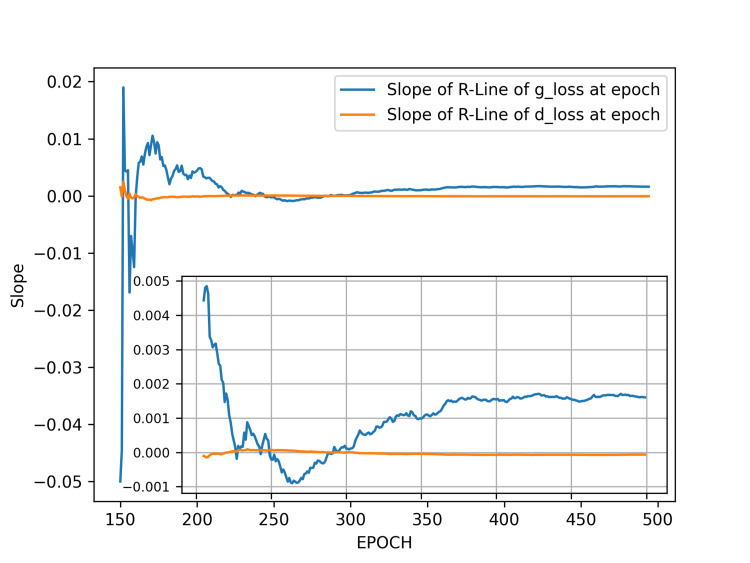
Slopes of the regression lines for generator and discriminator losses between epochs 155 to 500 in result 2. The plot displays the slopes across epochs from 155 to 500. The inset zooms in specifically on epochs 200 to 500 for a closer inspection. Between epochs 250 and 300 in result 2, the slopes of both g_loss and d_loss are slightly positive, indicating that the training process has reached a stable state. Abbreviations: g_loss, generator loss; d_loss, discriminator loss; R-Line, regression line.

Therefore, we excluded these particular values and started the regression analysis from epoch 150, as we believe this approach is more suitable for evaluating the optimal stopping point for training. Figure 6 shows the plot of the regression line slopes over epochs, based on data from epoch 155 to epoch 500.

Result 3

Similar to result 2, the generator loss steadily increases during the initial training phase, only reversing downward around epochs 100-120. However, unlike result 2, the generator loss begins to steadily increase again by epoch 400. We observed that, under these conditions, meaningful CXR images could not be generated, resulting instead in a high level of noise. If the loss trend is not recorded, this situation may give the false impression that training is ineffective.

After 500 epochs, the descriptive statistical parameters for the generator model’s training loss were as follows: mean = 4.778, median = 3.913, standard deviation = 1.691, minimum = 1.345, and maximum = 8.427. For the discriminator model’s training loss, the parameters were as follows: mean = 0.536, median = 0.540, standard deviation = 0.032, minimum = 0.501, and maximum= 0.905. We conducted a linear regression analysis on the generator and discriminator losses after completing 500 epochs, resulting in two linear equations: the generator loss is Y = 0.00099 X + 4.53168, and the discriminator loss is Y = -0.00002 X + 0.54083, where Y represents the respective regression value of the loss, and X represents the epoch.

**Figure 7:**
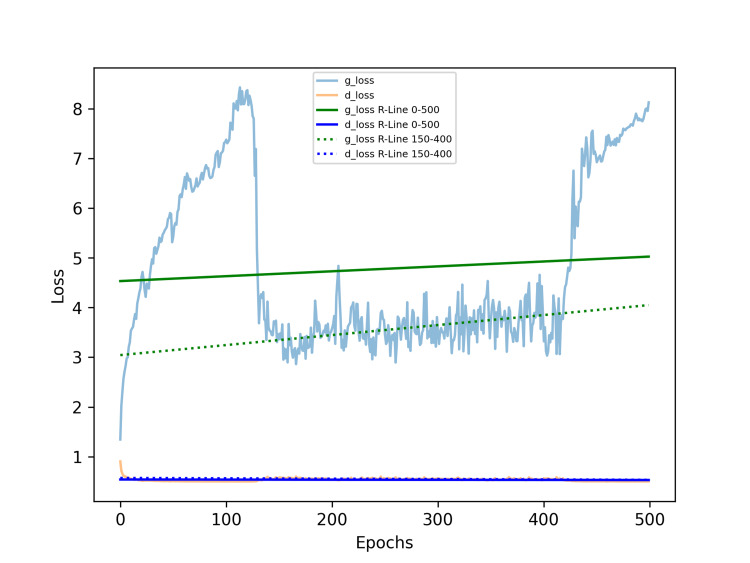
Loss curves and regression lines of the generator and discriminator in result 3. The g_loss gradually increased, peaking around 100-120 epochs and reversing to decrease, similar to result 2. After approximately the 150th epoch, the g_loss leveled out, fluctuating within a range of 3 to 4 and trending upward again until the 400th epoch. The d_loss, in contrast, remained relatively low and stable throughout the entire training. The green solid line represents the regression trend of g_loss over 500 epochs, while the blue solid line represents the regression trend of d_loss over the same period. The green dotted line shows the regression trend for g_loss from the 150th to the 400th epoch, and the blue dotted line represents the corresponding trend for d_loss during this same range. Abbreviations: g_loss, generator loss; d_loss, discriminator loss; R-Line, regression line.

These two lines were subsequently plotted on the loss history chart. We believe the values of the initial and later peaks do not contribute to our analysis; therefore, we conducted a linear regression analysis only on the losses from epochs 150 to 400 for both the generator and discriminator. The generator loss is Y = 0.00201 X + 3.04335, and the discriminator loss is Y = -0.00007 X + 0.57195. Refer to Figure 7.

**Figure 8:**
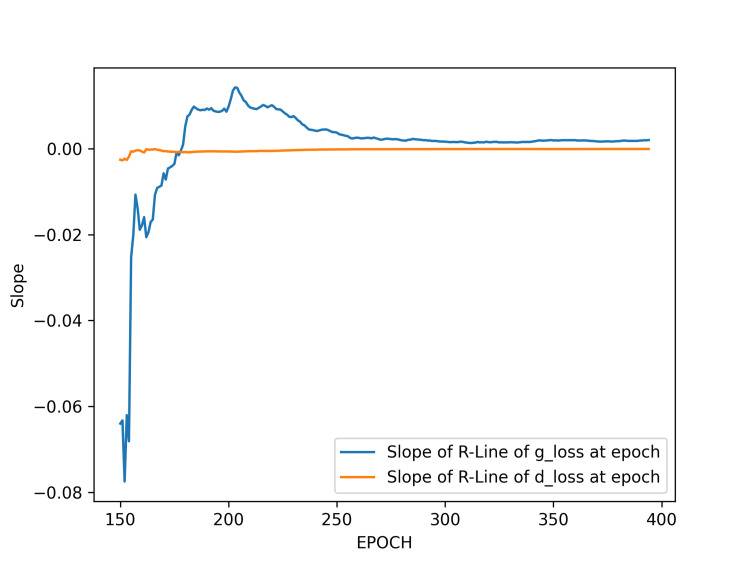
Slopes of the regression lines for generator and discriminator losses between epochs 155 to 400 in result 3. The plot displays the slopes across epochs from 155 to 500. Between epochs 250 and 300 in result 3, the slopes of both g_loss and d_loss are slightly positive, indicating that the training process has reached a stable state. Abbreviations: g_loss, generator loss; d_loss, discriminator loss; R-Line, regression line.

We excluded the loss values from epochs 0 to 150 and from 400 to the final epochs and began the slope analysis of regression over the range from epoch 155 to epoch 400. Figure 8 shows the plot of the regression line slopes over epochs, based on data from this range. We observe initial oscillations in the slope values, which quickly stabilize. Note that although the plot displays a pronounced peak in bi-directions, the actual values of this peak are not large.

## Discussion

There are three common types of generative AI models for image generation: diffusion model, variational autoencoder (VAE), and GAN. The diffusion model has gained significant attention for its ability to produce high-quality, detailed images. It operates by modeling the gradual corruption of data with noise and then learning to reverse this noise process to generate new data from random noise. Inspired by thermodynamic principles, the diffusion model is known for its stability and high-quality outputs, particularly in text-to-image generation [22]. VAE is a generative model that extends traditional autoencoders with probabilistic inference [23]. It is designed to generate new data that resembles a given training dataset. VAE is widely used in tasks such as image generation, anomaly detection, and data representation learning. GAN, introduced by Ian Goodfellow et al. in 2014 [24], is another class of generative models. GAN consists of two main components, the generator and the discriminator. The generator creates fake images, while the discriminator distinguishes between real and fake images. These two components compete with each other to continuously improve the image generation process.

In our study, we utilized a variant of GAN, the DCGAN, which was first introduced by Radford and colleagues in 2015 [21]. DCGAN employs convolutional layers to extract image features and deconvolutional layers for image generation. Compared to the original GAN architecture [24], it eliminates fully connected layers, reducing the number of parameters and preventing overfitting between the generator and discriminator. LeakyReLU is used as the activation function to mitigate the vanishing gradient problem and promote stable model training. Batch normalization also plays a crucial role in this architecture, enhancing the quality of the generated images.

DCGAN is widely applied in tasks such as image synthesis, style transfer, image restoration, and various other image-related applications. Notable examples include myocardial perfusion imaging in cardiology [25], blood flow prediction in laser speckle contrast imaging [26], conversion of T2-weighted MRI images of cervical spine trauma into short T1 inversion recovery images in radiology [27], and retinal fundus imaging in ophthalmology [28]. Additionally, DCGAN is used for image generation to expand the number of training datasets [29]. In medical fields, where data acquisition is subject to strict ethical reviews, patient consent [30], high costs [31], the need for strict regulation [32], and caution to avoid harm [33], DCGAN offers a potential solution to the shortage of training data.

In the field of CXR, limited use of DCGAN has been reported. Most articles on the application of DCGANs in CXR focus on data augmentation, which has been used for small sample sizes in training datasets [29] and for addressing imbalanced categories in datasets [34]. One study even suggested that GANs can detect COVID-19 in a semi-supervised setting [35]. Our research demonstrates that GANs are indeed capable of generating distinct features in synthetic CXRs, even at resolutions much lower than typical CXR images. These synthetic CXRs still retain recognizable macroscopic anatomical characteristics.

The generator is continually trying to fool the discriminator during training; however, it still has periods when it hardly produces images that can consistently fool the discriminator. That is the reason why a high variance in generator loss could happen in this plot of loss history during training, indicating instability in the adversarial training process. Around epoch 150 and beyond, the generator loss shows large spikes, which might be due to the discriminator becoming too strong, making it harder for the generator to improve.

There is no optimal training time for GANs, and in theory, the generator should improve over time. However, in our study, the generator loss diverged throughout the entire 500 epochs of training across three results. Additionally, through post-hoc regression analysis of the loss data at different intervals, we found that the most appropriate stopping time can be determined by examining the slope of the generator loss regression line. If the slope is from negative or downward-sloping to positive or near-horizontal, it indicates a suitable point to stop the training at that epoch.

On our hardware setup, training a model for grayscale images of size (256×256) using an NVIDIA RTX 3060 requires approximately 4,000 seconds for 500 epochs. However, if the training follows the pattern observed in result 3, this computational and energy-intensive effort may not yield meaningful results. Since GANs require substantial computational power, finding an appropriate training stopping point can save both computing resources and energy. Based on the discussions in this paper, we improved the training process by configuring the program to display generator and discriminator loss plots, as well as the regression line slope plot, every 10 training iterations. Additionally, the model is saved to disk every 50 iterations for later retrieval. This refinement prevents blindly running the training process to completion and enables stopping at an appropriate time without affecting the quality of the final generative output.

While DCGAN-generated images exhibit key features, their blurry and coarse quality limits their practical applications. Addressing this limitation may require deeper architectures and higher-resolution images. With advancements in more precise and sophisticated image generation, promising applications in medical education emerge, such as generating diverse diagnostic images for radiology exams and providing training materials for beginners in image interpretation.

However, ethical considerations surrounding the use of such technology must be carefully addressed. Regulations are needed to prevent the misuse of generated images, such as presenting them as authentic patient data in clinical settings without proper validation, which could mislead medical professionals or jeopardize patient safety.

Another critical concern is the safeguarding of patient privacy. Although generated images may not directly reproduce real patient data, precautions must be taken to prevent reverse engineering or the inadvertent embedding of identifiable patterns from training datasets. Establishing robust guidelines and regulatory frameworks will be essential to ensure the responsible use of generative models in medical applications, balancing innovation with ethical integrity.

Limitations

In this study, the generated CXRs primarily depict major anatomical structures because our dataset consists predominantly of normal CXRs, without including images of disease conditions such as pneumonia or pleural effusion. Consequently, the generated CXRs mostly exhibit normal findings. Moreover, for more detailed structures, such as precise tracheal anatomy, lung hilum, interstitial patterns, or nodular/mass lesions, our model was unable to generate them at the resolution available with our training setup. This limitation arises mainly from the use of a consumer-grade GPU, where the computational power and storage capacity of our local machine cannot support higher-resolution training. Lastly, the model lacks the ability to control the type of images generated, as the GAN produces outputs randomly. Unlike tools such as MidJourney or DALL-E, which can generate specific images based on prompts, this restricts its applicability for clinical purposes.

## Conclusions

In the application of generative imaging, DCGANs demonstrate the capability to produce diverse images that share common features. In our study, the medical images generated by GANs successfully preserved key characteristics. The final image outputs were acceptable in terms of gross anatomy; however, the resolution still requires improvement. This approach is not limited to CXRs and can be extended to other imaging modalities, such as CT or MRI. Compared to publicly accessible image models, the DCGAN model is smaller, can be trained locally at an affordable cost, and produces acceptable images. We propose a method for monitoring training progress, which could potentially serve as a reference standard for determining the optimal stopping point in training to prevent the production of erroneous results.
